# Safety, reactogenicity, and immunogenicity of a 2-dose Ebola vaccine regimen of Ad26.ZEBOV followed by MVA-BN-Filo in healthy adult pregnant women: study protocol for a phase 3 open-label randomized controlled trial

**DOI:** 10.1186/s13063-022-06360-3

**Published:** 2022-06-20

**Authors:** Etienne Karita, Julien Nyombayire, Rosine Ingabire, Amelia Mazzei, Tyronza Sharkey, Jeannine Mukamuyango, Susan Allen, Amanda Tichacek, Rachel Parker, Frances Priddy, Felix Sayinzoga, Sabin Nsanzimana, Cynthia Robinson, Michael Katwere, Dickson Anumendem, Maarten Leyssen, Malinda Schaefer, Kristin M. Wall

**Affiliations:** 1 Rwanda Zambia Health Research Group, Center for Family Health Research/Projet San Francisco, Kigali, Rwanda; 2 Rwanda Zambia Health Research Group, Department of Pathology & Laboratory Medicine, School of Medicine, Emory University, Atlanta, GA USA; 3 Madison, USA; 4 Rwanda Biomedical Center, Kigali, Rwanda; 5 Janssen Vaccines and Prevention, Leiden, the Netherlands; 6 Department of Obstetrics, Gynecology and Reproductive Sciences, Division of Maternal Fetal Medicine, University of Pittsburgh Magee-Women’s Hospital, Pittsburgh, PA USA; 7 Department of Epidemiology, Rollins School of Public Health, Laney Graduate School, Emory University, Atlanta, GA USA

**Keywords:** Ebola virus, Vaccine safety, Reactogenicity, Immunogenicity, Pregnancy

## Abstract

**Background:**

Risks to mother and fetus following Ebola virus infection are very high. Evaluation of safety and immunogenicity of non-replicating Ebola vaccine candidates is a priority for use in pregnant women. This is the protocol for a randomized, open-label, single-center phase 3 clinical trial of the safety, reactogenicity, and immunogenicity of the 2-dose Ebola vaccine regimen in healthy adult pregnant women. This 2-dose regimen has been shown to be safe, judged effective, and approved in non-pregnant populations.

**Methods:**

A total of 2000 adult (≥ 18 years of age) pregnant women will be enrolled from antenatal care facilities in Western Rwanda and randomized (1:1) to receive the 2-dose Ebola vaccine regimen (Ad26.ZEBOV, MVA-BN-Filo (group A)) or control (unvaccinated pregnant women (group B)). The primary objectives are to (1) assess adverse maternal/fetal outcomes in randomized pregnant women up to 1.5 months after delivery and (2) assess adverse neonatal/infant outcomes in neonates/infants born to randomized women up to 3.5 months after birth. The frequency and relatedness of all serious adverse events in women and newborns from randomization or birth, respectively, until study end will be reported. The reactogenicity and unsolicited adverse events of the 2-dose Ebola vaccine regimen in all vaccinated pregnant women (group A) will be reported. We will also assess the immunogenicity of the 2-dose Ebola vaccine regimen in 150 pregnant women who are anticipated to receive both vaccine doses within the course of their pregnancy (a subset of the 1000 pregnant vaccinated women from group A) compared to 150 non-pregnant women vaccinated after delivery (a subset of group B). The persistence of maternal antibodies in 75 infants born to women from the group A subset will be assessed. Exploratory analyses include assessment of acceptability of the 2-dose Ebola vaccine regimen among group A and assessment of maternal antibodies in breast milk in 50 women from group A and 10 controls (women from group B prior to vaccination).

**Discussion:**

This study is intended to support a label variation to relax restrictions on use in pregnant women, a vulnerable population with high medical need.

**Trial registration:**

Clinicaltrials.gov NCT04556526. September 21, 2020.

## Introduction

### Background and rationale {6a}

Ebola viruses spread in the human population through human-to-human transmission via direct contact (broken skin or mucous membranes) with blood, secretions, organs or other bodily fluids of infected persons, and with surfaces and materials (e.g., bedding and clothing) contaminated with these fluids. Ebola virus disease (EVD) has a mortality rate ranging from 40 to 90% according to the World Health Organization (WHO) [1].

The risks to mother and fetus following EVD infection during pregnancy are very high [2]. Prior to the 2013–2016 EVD epidemic, the case fatality rate for pregnant women was 89% (88/99) [3]. In the 2013–2016 epidemic, the case fatality rate was 53% (49/92). Pregnant women who survived EVD almost always lost their pregnancy, and survival among infants born to mothers with EVD is low [4]. Finally, infected mothers are a risk for infection of health care providers and other birth attendants.

Recently, a vaccine (Ervebo, Merck) was licensed for protection against EVD and this vaccine was used under an expanded access program during the EVD outbreak in Democratic Republic of Congo (DRC) that ended in 2020, including in pregnant women. Because Ervebo is a live replicating vaccine, there are some concerns with using it in pregnant women and therefore the WHO Strategic Advisory Group of Experts (SAGE) working group recommended in October 2018 “…, the development of non-replicating Ebola vaccine candidates should proceed with priority, as they represent fewer safety concerns for use in pregnancy” [5].

Janssen has developed a 2-dose heterologous Ebola vaccine regimen consisting of 1 dose of Ad26.ZEBOV vaccine, followed by 1 dose of MVA-BN-Filo vaccine 8 weeks later, for active immunization for prevention of disease caused by Ebola virus (Zaire ebolavirus species) which is approved for individuals ≥ 1 year of age. Both Ad26.ZEBOV and MVA-BN-Filo are replication-incompetent vectored vaccines [6].

On 27 September 2019, the Rwanda FDA granted the Janssen Ebola vaccine conditional approval under exceptional emergency circumstances to facilitate a large vaccination campaign (Umurinzi) that started on 8 December 2019 and aims to vaccinate up to 200,000 non-pregnant Rwandans aged 2 years and older in the Western districts bordering with DRC. As of 14 September 2021, at least 200,000 people have received both doses.

#### Clinical efficacy/safety studies in non-pregnant populations

The safety, reactogenicity, and immunogenicity of the Ad26.ZEBOV and MVA-BN-Filo vaccines have been/are being evaluated in completed and ongoing clinical studies in adults and children ≥ 1 year of age. Janssen’s current clinical development plan contains 4 completed phase 1 studies (EBL1001 [7] EBL1002 [8], EBL1003 [9], EBL1004 [10, 11]), 1 completed phase 2 study (EBL2001) (manuscript under review), 2 completed phase 3 studies (EBL3002 and 3003) (manuscript in preparation), 3 completed phase 2/3 studies (EBL2002, EBL2003, EBL3001), and an ongoing phase 2/3 study that has completed dosing and is in the follow-up phase (EBL4001) (manuscript under review). Unblinded safety data from 2390 adults from studies (EBL1001, EBL1002, EBL1003, EBL1004, EBL2001, EBL2002, EBL3001, EBL3002, EBL3003, and FLV1001), including 1814 healthy and 118 HIV + adults dosed with Ad26.ZEBOV, MVA-BN-Filo [*N* = 1932], and 434 healthy and 24 HIV + adults dosed with control [placebo or active control, *N* = 458] is summarized. Overall, the safety profile consists of mild to moderate AEs of short duration with no sequelae, confirming results of the phase 1 studies. No safety signals were identified. The frequency of grade 3 pyrexia (≥ 39.0 °C) was < 1% following vaccination, and the incidence of any febrile response was < 7.5% in any group. SAEs were reported in 54 participants (2.8%) vaccinated with the active vaccine regimen and in 11 participants (2.4%) vaccinated with control.

The immunogenicity of the Ad26.ZEBOV, MVA-BN-Filo vaccine regimens was also confirmed in phase 2 and 3 studies. Because the 2013–2016 Ebola outbreak subsided before clinical efficacy data could be generated, it was agreed with regulators (the European Medicines Agency [EMA] and the United States Food and Drug Administration [FDA]) to infer the protective effect of the vaccine from immunobridging studies [12]. Data from 5 clinical studies conducted in Europe, the USA, and Africa in 764 adults 18 to 50 years of age who had received the 2-dose regimen at the 8-week interval were used in this analysis. Based on this analysis, the Ad26.ZEBOV, MVA-BN-Filo vaccine regimen can be anticipated to have a protective effect against EVD in humans.

#### Pre-clinical safety data in pregnancy

A pre-clinical study with Ad26.ZEBOV, MVA-BN-Filo regimens (TOX11212) conducted in young adult New Zealand White rabbits was conducted to determine maternal and developmental toxicity following maternal exposure to the vaccine regimens. The Ad26.ZEBOV and MVA-BN-Filo regimens did not induce maternal or developmental toxicity following maternal exposure during the premating and gestation period. The vaccine regimen elicited detectable EBOV GP-specific maternal antibody titers that were transferred to the fetuses.

#### Vaccination during pregnancy

Pregnancy was an exclusion criterion for the past clinical trials in this program. A mandatory pregnancy test was performed before each vaccination and a commitment to use adequate contraception was requested from all females of childbearing potential. A minimal number of inadvertent pregnancies occurring during clinical trials with the Ebola candidate vaccines Ad26.ZEBOV, Ad26.Filo, and MVA-BN-Filo have been followed to term.

The most recent aggregate review of pregnancy exposure data was performed in August 2019. This analysis of the current experience with pregnancies after exposure to the Ebola candidate vaccines (Ad26.ZEBOV, Ad26.Filo, MVA-BN-Filo) in female study participants or partners of male study participants did not reveal a safety concern.

Serious complications/SAEs during pregnancy were reported in 24 out of a total of 79 pregnancies, i.e., in 20 women study participants and in 4 women partners of male study participants. None of these serious complications/SAEs were considered causally associated with the study vaccines by investigators and/or the company. No apparent concerning pattern of AEs is emerging from this review. No congenital malformations were reported to date to the company in fetuses or newborns. Spontaneous abortion was the most commonly observed SAE (11 out of 79 pregnancies) with an incidence of 13.9%, which is within the range of expected spontaneous abortion rates during the first trimester of gestation even when considering that spontaneous abortion incidences vary significantly depending on geographical areas and individual risk factors (e.g., age, previous abortions) [13–16].

#### Benefit-risk assessment related to study participation

Data from the minimal number of inadvertent pregnancies discussed above is insufficient to establish vaccine safety in pregnant women or neonates or immunogenicity in pregnant women, and the theoretical risk of the vaccine administered on the safety of mother and infant is unknown. However, it is expected that no concerning safety signal will occur for mothers or neonates given that (1) both Ad26.ZEBOV and MVA-BN-Filo are non-replicating vaccines and are not expected to be transferred trans-placentally to infect the fetus after intramuscular (IM) vaccination; (2) their limited biodistribution has been confirmed in animal studies; (3) a pivotal combined embryofetal and pre-and postnatal developmental study in rabbits did not indicate a risk of vaccination to the fetus; and (4) limited available data on inadvertent exposure to the vaccines during pregnancy also did not raise any safety concern, including for vaccinations delivered during the first trimester.

The immune response of pregnant women has not been extensively studied by trimester. Despite a trend toward lower immune responses in the first trimester and 6 weeks post-partum, Sperling et al. [17] did not find evidence that timing of vaccination significantly altered immune response to a monovalent H1N1 vaccine administered to 239 women during pregnancy or post-partum. Post vaccination anti-EBOV GP antibodies peak about 21 days post dose 2 and persist, with some waning, over a 1–2-year period. Fetuses/embryos in women vaccinated during earlier trimesters may be exposed to maternal antibodies for longer periods allowing more robust transfer of maternal antibodies trans-placentally. However, trans-placental antibody transfer generally gradually increases as pregnancy progresses [18, 19], and immunization of pregnant women is generally recommended in the second or third trimester to maximize the potential of passive transfer of maternal antibodies to the fetus. IgG transfer from the mother to the fetus can occur starting from gestational week 13 (end of the first trimester), although the majority of IgG transfer occurs during the third trimester. Additional forms of passive immune transfer occur postnatally and involve vaccine-induced IgA, IgG, and IgM being secreted into the colostrum and breast milk. There is evidence that maternal vaccination can inhibit neonatal humoral immune response to vaccination. However, this interference mainly impacts primary immunization and not subsequent booster immunizations. These topics are reviewed in Faucette et al. [20].

Based on the available data and proposed safety measures, the overall benefit/risk assessment for this clinical study is considered acceptable for the following reasons: (1) to date, safety data from the studies in the clinical development program revealed no safety issues of concern. Further experience from Ad26.ZEBOV or MVA-BN-Filo will be gained from currently ongoing clinical studies; (2) the selection criteria include adequate provisions to minimize the risk and protect the well-being of participants and their fetuses/neonates/infants in the study; (3) safety will be closely monitored throughout the study; (4) several safety measures are included in this protocol to minimize the potential risk to participants. In addition to the potential protection against Ebola from the vaccine, participants and their newborns may benefit from close medical follow-up, including clinical testing and physical examination, during pregnancy, at delivery, up to 6 weeks post pregnancy and 14 weeks of age for infants. Others may benefit from the knowledge that they may aid in the development of an EVD vaccine.

### Objectives {7}

The objective of the proposed study is to evaluate the safety, reactogenicity and immunogenicity of a 2-dose Ebola vaccine regimen of Ad26.ZEBOV followed by MVA-BN-Filo in healthy pregnant women. No formal statistical hypothesis is to be tested. The study is designed to provide descriptive information regarding the rates of adverse maternal/fetal and neonatal/infant outcomes after administration of the 2-dose Ebola vaccine regimen (Ad26.ZEBOV, MVA-BN-Filo) during pregnancy. This study is intended to support a label variation of the relevant sections of the prescribing information, to relax restrictions on use in pregnant women, a vulnerable population with high unmet medical need.

### Trial design {8}

This is a randomized, open-label, single center phase 3 clinical trial of the safety, reactogenicity, and immunogenicity of the 2-dose Ebola vaccine regimen of Ad26.ZEBOV followed by MVA-BN-Filo in healthy adult pregnant women.

## Methods: participants, interventions, and outcomes

### Study setting {9}

The study will be conducted at the government-run Gisenyi District Hospital in Rubavu, Rwanda, and the Gihundwe District Hospital in Rusizi, Rwanda. These District Hospitals are in the Western Province of Rwanda that borders the DRC and are the areas of Rwanda at highest risk of EVD. This is the same location as the Umurinzi campaign.

### Eligibility criteria {10}

#### Inclusion criteria

1. Female (according to their reproductive organs and functions assigned by chromosomal complement)
2. 18 years of age or older at the time of written informed consent
3. Healthy on the basis of physical examination, medical history, obstetric history, and vital signs performed at screening
4. Healthy on the basis of clinical laboratory tests performed at screening. If the results of the laboratory screening tests are outside the normal local reference ranges, the participant may be included only if the investigator judges the abnormalities or deviations from normal to be not clinically significant or to be appropriate and reasonable for the population under study. This determination must be recorded in the participant’s source documents and initialed by the investigator. Trace protein in the urine is acceptable if the blood pressure is also normal. Participants must have screening laboratory tests results within the following parameters:
  - Hemoglobin ≥ 7.0 g/dL
  - White blood cells ≥ 3.4 × 10^3^ cells/μL
  - Neutrophils ≥ 1.1 × 10^9^ cells/L
  - Lymphocytes ≥ 1.21 × 10^9^ cells/L
  - Platelets ≥ 156 × 10^3^ cells/μL
  - Serum creatinine ≤ 129 μmol/L
  - Aspartate aminotransferase (AST) within 1.5 times the upper limit of normal range for the laboratory conducting the test
  - Alanine aminotransferase (ALT) within 1.5 times the upper limit of normal range for the laboratory conducting the test
  - Total bilirubin ≤ 25 μmol/L
  - Urine protein < 1 + by dipstick
  - Urine blood ≤ 1 + by dipstick, without evidence of bacteriuria on microscopy
5. Confirmed singleton pregnancy by positive urine HCG and ultrasound at time of screening and informed consent and reconfirmed pregnancy via ultrasound at randomization/day 1. Ultrasound not required on randomization/day 1 if ≤ 10 days have elapsed since screening ultrasound and ultrasound is not indicated for other reasons
6. Capable and willing to give informed consent (signed or thumbprint) which includes compliance with the requirements and restrictions listed in the ICF and in this protocol and is willing to give consent for the infant to participate in the study
7. Each potential participant must pass the AOU indicating that she understands the purpose, procedures and potential risks and benefits of the study, after reading the informed consent and after the investigator or designee has provided detailed information on the study and has answered the potential participant’s questions. Each participant must subsequently sign the ICF, indicating that she is willing to participate in the study
8. Residing within catchment area of the study site
9. Able and willing to participate for the duration of the study visits and follow-up
10. Willing and able to comply with the protocol requirements
11. Willing to receive standard prenatal care and planning to deliver at a study District Hospital
12. Willing to provide verifiable identification, have a photo taken, and iris scan at study entry and follow-up visits
13. Evidence of normal progress of gestation prior to randomization (day 1) based on obstetric evaluation (including obstetric history, obstetric examination and fetal ultrasound)
14. If randomized to group B, must have negative urine pregnancy test immediately prior to each study vaccine administration

#### Exclusion criteria

1. History of EVD (self-declared or laboratory confirmed)
2. Has received any candidate Ebola vaccine (except for women found to be pregnant in the Umurinzi study after they receive their first vaccine dose, who are eligible for enrollment into a non-randomized group under this protocol)
3. Has received any experimental candidate Ad26- or MVA-based vaccine in the past. Receipt of any approved vaccinia/smallpox vaccine or Ad-based candidate vaccine other than Ad26 at any time prior to study entry is allowed
4. Known allergy or history of anaphylaxis or other serious adverse reactions to vaccines or vaccine products (including any of the constituents of the study vaccines [e.g., polysorbate 80, ethylenediaminetetraacetic acid (EDTA) or L-histidine for Ad26.ZEBOV vaccine; tris (hydroxymethyl)-amino methane (THAM) for MVA-BN-Filo vaccine]), including known allergy to egg, egg products, chicken proteins, and aminoglycosides
5. Participant with acute illness (this does not include minor illnesses such as diarrhea or mild upper respiratory tract infection) or body temperature ≥ 38.0 °C on day 1 will be excluded from enrollment at that time but may be rescheduled for enrollment at a later date
6. Presence of significant conditions (e.g., history of seizure disorders, (auto)immune disease or deficiency, any spleen disease, active malignancy, ongoing tuberculosis treatment, other systemic infections) or clinically significant findings during screening of medical history, obstetric history, physical examination, vital signs or laboratory testing for which, in the opinion of the investigator, participation would not be in the best interest of the participant (e.g., compromise the safety or well-being) or that could prevent, limit, or confound the protocol-specified assessments. Participant who have recently received treatment for acute, uncomplicated malaria are eligible for participation if at least 3 days have elapsed from the conclusion of a standard, recommended course of therapy for malaria; subjects who are acutely ill with malaria at the time of screening should complete therapy and wait an additional 3 days after completion before screening for the study. Subjects with sickle cell trait can be included
7. During the 6 weeks prior to screening, have had ANY of (a) confirmed SARS-CoV-2 (COVID-19) infection (test positive), OR (b) suspected SARS-CoV-2 infection (clinical features without documented test results), OR (c) close contact with a person with known or suspected SARS-CoV-2 infection
8. History of or underlying liver or renal insufficiency, or significant cardiac, vascular, pulmonary (e.g., persistent asthma), gastrointestinal, endocrine, neurologic, hematologic, rheumatologic, psychiatric, or metabolic disturbances
9. History of hepatitis B surface antigen (HBsAg) or hepatitis C antibody (anti-HCV) positive, or other clinically active liver disease, or tests positive for HBsAg or anti-HCV at screening
10. HIV-infected but not on stable antiretroviral therapy, defined as adherent to ART regimen for ≥ 6 weeks prior to enrolment. HIV-infected subjects on stable ART may be enrolled
11. Obstetric history including:
  a  ≥ 2 consecutive spontaneous abortions
  b History of pre-eclampsia or eclampsia
  c Rhesus negative multigravida
  d Grand multigravida (> 5 previous pregnancies)
  e Previous late still birth (defined as loss of pregnancy at any time after 28weeks gestation)
  f Previous low birth weight baby or premature delivery (defined as a delivery before 37weeks gestation)
  g Previous neonatal death (defined as death of an infant within the first 28days of life)
  h Previous delivery of an infant with a known or suspected genetic or chromosomal abnormality
  i History of other significant pregnancy-related or neonatal complications judged likely to affect the safety of the mother or infant or to significantly compromise the endpoint data collected. Previous cesarean section is not an exclusion criterion
12. Major surgery within the 4 weeks prior to screening or planned major surgery through the course of the study (from screening until completion of the study)
13. Chronic or recurrent use of immunomodulators/suppressors, e.g., cancer chemotherapeutic agents, systemic corticosteroids within 6 months before the planned administration of the first dose of study vaccine. Ocular, topical or inhaled steroids are allowed
14. Received or plan to receive a licensed non-live attenuated vaccine (e.g., tetanus) within 7 days of a study vaccination (i.e., before or after). Received or plan to receive licensed live attenuated vaccines within 4 weeks of a study vaccination (i.e., before or after)
15. Received an investigational drug or investigational vaccine or used an invasive investigational medical device within 3 months prior to screening or current or planned participation in another clinical study during the study. Participation in an observational clinical study is allowed
16. Receipt of blood products or immunoglobulin within 3 months prior to screening and/or during participation in the study (with the exception of RhoGAM)
17. Current or past abuse of alcohol, recreational, or narcotic drugs which in the investigator’s opinion would compromise the subject’s safety and/or compliance with the study procedures
18. History of chronic urticaria (recurrent hives)
19. Participant cannot communicate reliably with the investigator
20. Employee of the investigator or study site, with direct involvement in the proposed study or other studies under the direction of that investigator or study site, as well as family members of the employees or the investigator
21. History of thrombotic thrombocytopenia syndrome (TTS) or heparin-induced thrombocytopenia and thrombosis (HITT)

### Who will take informed consent? {26a}

A study investigator or an authorized member of the study-site personnel will obtain informed consent from trial participants. Before enrollment in the study, the investigator or an authorized member of the study-site personnel must explain to potential participants the aims, methods, reasonably anticipated benefits, and potential hazards of the study and any discomfort participation in the study may entail. Participants will be informed that their participation is voluntary and that they may withdraw consent to participate at any time. They will be informed that choosing not to participate will not affect the care the participant will receive. Finally, they will be told that the investigator will maintain a participant identification register for the purposes of long-term follow-up if needed and that their records may be accessed by health authorities and authorized sponsor personnel without violating the confidentiality of the participant, to the extent permitted by the applicable law(s) or regulations. Because many women in this area are unable to read or write, the informed consent will be presented as a video. The video will be recorded in the local language, Kinyarwanda. The video will be paused at frequent points to allow for discussion and questions.

Each potential participant must pass the Assessment of Understanding indicating that she understands the purpose, procedures, and potential risks and benefits of the study, after reading the informed consent and after the investigator or designee has provided detailed information on the study and has answered the potential participant’s questions. Potential participants are allowed to retake the test twice to achieve the passing score (≥ 90%) required for participation in the study. If a potential participant fails to achieve the passing score, further information and counseling needs to be provided by the study team member after each failed attempt. Any potential participant not capable of understanding the key aspects of the study, and their requirements for participation, should not be enrolled.

Consent should then be appropriately recorded by means of the participant’s personally dated signature or thumbprint. After having obtained the consent, a copy of the ICF must be given to the participant. Participants who are rescreened are required to sign a new ICF. Informed consent for follow-up of the newborn after day of life 28 will be obtained from the mother at the time of screening.

### Additional consent provisions for collection and use of participant data and biological specimens {26b}

A total of 300 pregnant women will be included in a sub-study of the immune response to the vaccine. From the mother, we will collect samples of blood, cord blood, and breast milk. We will also seek permission to collect a small sample of blood from 75 babies [born to mothers that receive study vaccine during pregnancy] when they are 3 months old. We will ask permission to keep any remaining samples for future studies.

## Interventions

### Explanation for the choice of comparators {6b}

The comparator group is unvaccinated pregnant women (group B). Robust data on background rates of adverse pregnancy outcomes are not available. Therefore, the unvaccinated pregnant women in group B (a non-interventional control) are important to establish the frequency and magnitude of changes in clinical endpoints that may occur in the absence of active vaccination during pregnancy (group A). Group B comparator women will receive the study vaccine after pregnancy termination.

### Intervention description {11a}

The Ebola vaccine regimen in this trial is comprised of two vaccines:

1. Ad26.ZEBOV is a replication-incompetent monovalent vaccine encoding the full-length glycoprotein (GP) from Ebola virus (EBOV) Mayinga. A 0.5-mL injection into the deltoid muscle in the upper arm (or thigh if needed) of 5 × 10^10^ vp will be administered at day 1 (enrollment).
2. MVA-mBN226B, further referred to as Modified Vaccinia Ankara (MVA)-BN®-Filo, does not replicate in human cells and is a multivalent vaccine encoding the EBOV GP, the Sudan virus (SUDV) GP, the Marburg virus (MARV) Musoke GP, and the Taï Forest virus (TAFV, formerly known as Côte d’Ivoire ebolavirus) nucleoprotein (NP). A 0.5 mL injection into the deltoid muscle in the upper arm (or thigh if needed) of 1 × 10^8^ InfU will be administered at day 57 (− 14 days + 28 days) after the Ad26.ZEBOV dose.

The second dose of vaccine should ideally be given in the opposite arm/thigh (unless the opposite arm/thigh has a condition that prevents evaluation of the arm/thigh after injection). The Ad26.ZEBOV and MVA-BN-Filo vaccines will be manufactured and provided under the responsibility of Janssen Vaccines and Prevention B.V.

### Criteria for discontinuing or modifying allocated interventions {11b}

Participants will be withdrawn from study vaccine administration for the reasons listed below. These participants must not receive any additional dose of study vaccine but should enter the follow-up phase with assessments of safety and immunogenicity. Dose modification is not applicable in this study.

1. Any related AEs, worsening of health status or intercurrent illnesses that, in the opinion of the investigator, required discontinuation from study vaccine
2. Confirmed EVD
3. Anaphylactic reaction following vaccination
4. Injection site ulceration, abscess, or necrosis considered to be related to study vaccine
5. Generalized urticaria within 72 h of vaccination considered to be related to study vaccine
6. SAEs or other potentially life-threatening (grade 4) event that is determined to be related to study vaccine
7. Chronic or recurrent use of immunosuppressants (after discussion with the sponsor)

### Strategies to improve adherence to interventions {11c}

To reduce the chances of a participant being lost to follow-up, prior to randomization attempts should be made to obtain contact information from each participant. The following actions must be taken if a participant fails to return to the study site for a required study visit:

- The study-site personnel must attempt to contact the participant to reschedule the missed visit as soon as possible, to counsel the participant on the importance of maintaining the assigned visit schedule, to ascertain whether the participant wishes to or should continue in the study
- Before a participant is deemed lost to follow-up, the investigator or designee must make every reasonable effort to regain contact with the participant (where possible, 3 telephone calls, e-mails, and/or SMS/text, or local equivalent methods). These contact attempts should be documented in the participant’s medical records
- Should the participant continue to be unreachable, they will be considered to have withdrawn from the study

Women will receive 12,000 Rwanda Francs for each study visit, equal to roughly $12 USD.

### Relevant concomitant care permitted or prohibited during the trial {11d}

Use of any experimental medication (including experimental vaccines other than the study vaccine) during the study is not allowed. Vaccination with licensed live attenuated vaccines within 4 weeks of a study vaccination (i.e., before or after) is prohibited. Non-live, licensed vaccines (e.g., tetanus, hepatitis A, hepatitis B, rabies) should be given at least 7 days before or 7 days after administration of study vaccine in order to avoid potential confusion of adverse reactions and potential immune interference. If a vaccine is indicated in a post-exposure setting (e.g., rabies or tetanus), it must take priority over the study vaccine. Chronic (> 10 days) or recurrent use of immunomodulators/suppressors, e.g., cancer chemotherapeutic agents, systemic corticosteroids is prohibited during the study and 6 months before the planned administration of the first dose of study vaccine. Ocular, topical, or inhaled steroids are allowed.

### Provisions for post-trial care {30}

Since this trial is enrolling healthy pregnant female participants for which the study vaccine is envisaged to be prophylactic, post-trial care will entail medical follow-up of any continuing SAEs until resolution or stabilization. In the event of trial-related harm or injury, medical care and follow-up will be arranged for affected trial participants at no cost to them. Clinical trial insurance has also been procured for this trial in accordance with national regulations in the participating country (Rwanda).

### Outcomes {12}

As described in Table 1, primary outcomes will be adverse maternal, fetal, and neonatal/infant outcomes in all pregnant women and their neonates/infants. Secondary outcomes will be the description of all serious AEs occurring in all pregnant women and their neonates and the description of all solicited (reactogenicity) and unsolicited AEs (up to 28 days following each vaccination) in all pregnant women from group A. The Brighton Collaboration case definitions for specific adverse maternal/fetal and neonatal outcomes will be used as recommended to provide robust safety evaluation which can be utilized across studies and populations. In a subset of 300 women from group A (*N* = 150) and group B (*N* = 150), immunogenicity will be assessed as secondary outcome. Additionally, persistence of maternal antibodies will be assessed as a secondary outcome in a convenience sample of 75 infants born to group A women in the immunogenicity subset. As an exploratory outcome, if an assay becomes available the presence of antibodies will be assessed in breastmilk in a convenience sample of 50 women from the group A immunogenicity subset who are able to express breastmilk at the time point indicated in the schedule of activities and 10 control women (women in group B before vaccination). Analysis metrics, method of aggregation, and time point for analysis of each outcome are described in the “Statistical methods” section.

**Table 1:** Trial objectives and endpoints

Objectives	Endpoints
**Primary**	
• Assess **adverse maternal/fetal outcomes in pregnant women** randomized to receive the 2-dose Ebola vaccine regimen (Ad26.ZEBOV, MVA-BN-Filo (group A)) and in control women (unvaccinated pregnant women (group B))	• Frequency of maternal death, spontaneous abortion, stillbirth, pathways to preterm birth (premature preterm rupture of membranes, preterm labor, insufficient cervix, provider initiated preterm birth), pre-eclampsia/eclampsia, antenatal bleeding and post-partum hemorrhage from randomization until 6 weeks post-completion/termination of pregnancy
• Assess **adverse neonatal/infant outcomes in neonates/infants** born to women randomized to receive the 2-dose Ebola vaccine regimen (Ad26.ZEBOV, MVA-BN-Filo (group A)) and in neonates/infants born to control women (unvaccinated pregnant women (group B))	• Frequency of major congenital malformations, small for gestational age (SGA), low birth weight, preterm birth, neonatal death, and failure to thrive in infants measured from birth until 14 weeks of age
**Secondary**	
• Assess safety **in pregnant women** randomized to receive the 2-dose Ebola vaccine regimen (Ad26.ZEBOV, MVA-BN-Filo group A) and in control women (unvaccinated pregnant women (group B))	• Frequency and relatedness of all SAEs in women from randomization until study end
• Assess safety **in neonates/infants born** to women randomized to receive the 2-dose Ebola vaccine regimen (Ad26.ZEBOV, MVA-BN-Filo (group A)) and in neonates/infants born to control women (unvaccinated pregnant women (group B))	• Frequency and relatedness of all SAEs in the newborns from birth until study end
• Assess the **reactogenicity** and unsolicited AEs of the 2-dose Ebola vaccine regimen (Ad26.ZEBOV, MVA-BN-Filo) in all vaccinated pregnant women (group A)	• Reactogenicity, defined as local and systemic solicited AEs occurring within 7 days after each dose, and unsolicited AEs within 28 days after each dose: ○ Frequency, grade, duration and causality for solicited systemic AEs and unsolicited AEs ○ Frequency, grade and duration for solicited local AEs
• Describe all pregnancy outcomes	• Pregnancy outcomes (example: normal delivery, Caesarian Section)
• Assess the **immunogenicity** of the 2-dose Ebola vaccine regimen (Ad26.ZEBOV, MVA-BN-Filo) in 150 pregnant women who are anticipated to receive both vaccine doses within the course of their pregnancy (a subset of the 1000 pregnant vaccinated women from group A) compared to 150 non-pregnant women vaccinated after delivery (a subset of group B)	• Anti-EBOV GP binding antibody concentrations, in ELISA units/mL (FANG ELISA) from: ○ Blood samples taken at pre-dose 1, 21 days post-dose 2, at delivery (group A subset only), and 1 year post-dose 1 ○ Cord blood if feasible from women in the group A subset
• Assess **persistence of maternal antibodies** in 75 infants born to women from the group A subset	• Anti-EBOV GP binding antibody concentrations, in ELISA units/mL (FANG ELISA) from a blood sample taken at 14 weeks of age
**Exploratory**	
• Assess acceptability of an Ebola vaccine among healthy pregnant women (group A)	• Description of vaccine acceptability among vaccinees after they receive both doses • Evaluation of factors associated with study enrollment (defined as the proportion of eligible women who agree to sign the informed consent)
• Assess presence of maternal antibodies in breast milk in 50 women from group A and 10 controls (women from group B prior to vaccination)	• Evaluation of Anti-EBOV GP binding antibodies depending on availability of assays; in a sample of breast milk at 6 weeks post-delivery

### Participant timeline {13}

See Tables 2, 3, 4, and 5 for the participant schedule of activities and visit timeline.

**Table 2:** Schedule of activities—women in group A

Visit #	1	2	3	4	5	6	7 ^a^	8	9	10	11^a^	EE
**Visit timing**^**p**^		**Dose 1**	**Dose 1** ** + 7 days**	**Dose 1 + ** **28 days**	**Dose 2**	**Dose 2 + ** **7 days**	**Dose 2 + ** **21 days**	**Dose 2 + ** **28 days**	**Pregnancy completion/** **termination**	**PP 1 + 42**	**Dose 1 + ** **365 days**	**Early** **Exit**
**Visit day and window**	** − 28 to 1**	**D1**	**D8 ± 3 days**	**D29 ± 7 days**	**D57** ** − 14 days, + 28 days**	**D64 ± 3 days**	**D78 ± 3 days**	**D85 ± 7 days**	**PP 1** ^b^ ** + 2 days**	**PP 43** ^c^ ** ± 14 days**	**Dose 1 + 365 ± 28 days**	
**Visit type**	Screening	**DOSE 1 group A**	Safety	Safety	**DOSE 2 group A**	Safety	Immuno	Safety	Safety and immuno	Safety	Safety and immuno	Early exit
Assessment of understanding	•											
Written informed consent (ICF) ^d^	•											
Inclusion/exclusion criteria	•	•										
Demographics ^**e**^	•											
Medical and obstetric history ^f^; pre-pregnancy and pre-study meds	•											
Physical examination ^f^	•				•					•		•
Obstetric exam ^f^	•	•			•				•			•^o^
Targeted physical exam ^f^		•	•	•		•	•	•			•	
Vital signs ^f^ incl. body temperature	•		•	•		•	•	•	•	•	•	•
Urine pregnancy test ^g^	•											
Hematology, chemistry ^g^	•											
Urine dipstick/urinalysis ^g^	•	•		•	•			•				
Syphilis, HIV, hepatitis B and C, malaria screening^g^	•											
Obstetric ultrasound ^h^	•	•^h^						•				•^o^
Biometric scan	•	•	•	•	•	•	•	•	•	•	•	•
Contact information	•	•	•	•	•	•	•	•	•	•	•	•
Randomization		•										
Pre-vaccination symptoms ^i^												
Vaccination		•			•							
30 min post-vaccination observation ^j^		•			•							
Solicited AE collection ^k^		•	•		•	•						
Unsolicited AE collection ^k^		•	•	•	•	•	•	•				
Distribution of participant diary, rulers and thermometers ^k^		•			•							
Participant diary review by site staff			•			•						
Adverse maternal, fetal outcomes		•	•	•	•	•	•	•	•	•		
SAE recording		•	•	•	•	•	•	•	•	•	•	•
Concomitant medications ^l^	•	•	•	•	•	•	•	•	•	•	•	•
Delivery history ^m^									•			
Post pregnancy physical examination ^m^									•			
Laboratory assessments^n^									•			
Acceptability questionnaire									•	•		•
Group A immunology subset supplemental procedures (*N* = 150)	**1**	**2**	**3**	**4**	**5**	**6**	**7**	**8**	**9**	**10**	**11**	
Humoral immunogenicity sample (serum)							•		•			
Cord blood sample									•		•	
Breast milk sample (*N* = 50)										•		

pre-dose;  pre- and post-dose

^a^Visits 7 and 11 for group A immunology subset participants only

^b^PP 1 refers to the day of pregnancy completion/termination. Visit 9 (PP 1) should occur on the day of pregnancy completion/termination or within 2 days

^c^Visit 10 (PP 43) scheduled to occur at routine 6 week post-partum visit. For women from group A not part of the immunogenicity subset, this visit will be the end of the trial

^d^Must be signed before first study-related activity

^e^Maternal demographic information to be collected at screening includes date of birth, race/ethnicity, education level, geographical location/residence, occupation, household size (number of persons living in the same home), and international travel history

^f^Information to be collected for medical and obstetric history, physical exams, and vital signs is specified in the protocol

^g^Urine dipstick for protein and glucose will be performed at these visits. If results 1 + or greater, then additional work-up for pre-eclampsia or gestational diabetes may be warranted

^h^Ultrasound not required at visit 2 if screening (visit 1) ultrasound completed within past 10 days and no other indication for ultrasound

^i^Investigator must check for acute illness or body temperature ≥ 38.0 °C at the time of vaccination. In such cases, the participant may be vaccinated at later time point

^j^Participants will be closely observed for a minimum of 30 min post-vaccination

^k^In all women of group A, solicited AEs (reactogenicity) and unsolicited AEs will be assessed 7 days post each dose and 28 post each dose respectively

^l^Includes any medical treatment/medications given to the mother during delivery (e.g., antibiotic prophylaxis)-specifying names of medications administered

^m^Information to be collected for delivery history and post-pregnancy examination is specified in the protocol

^n^Any routine laboratory assessment performed at time of delivery and other laboratory tests as medically indicated

^o^Not required if participant is not pregnant

^p^In case pregnancy completion/termination in a group A pregnant woman occurs prior to receipt of dose 2, visits 9 and 10 should be performed: visit 10 being relative to visit 9 (2 day window). Visit 5 (dose 2 vaccination with a window of – 14 days, + 28 days) should in this case be performed after visit 9, with visits 6 and 8 being relative to actual dose 2 receipt (visit 5). All of these visits should be captured in RedCap cloud in addition to source documentation. Reactogenicity data (diary completion) should still be performed following dose 2 for group A women that have a pregnancy completion/termination prior to receipt of dose 2

**Table 3:** Schedule of activities—women in group B

Visit #	1	2	3	4	5	6	7	8	9^a^	10^n^	11 ^a^	EE
**Visit timing**^**p**^		**Randomiz-ation (D1)**	**D1 + 28 days**	**D1 + 56 days**	**D1 + 12 weeks**	**Pregnancy completion/** **termination**	**Dose 1**	**Dose 2**	**Dose 2 + ** **21 days**	**Dose 2 + ** **28 days**	**Dose 1 + ** **365 days**	**Early** **exit**
**Visit day and window**	**-28 to 1**	**D1**	**D29 ± 7 days**	**D57** ** − 14 days, + 28 days**	**D85 ± 7 days**	**PP 1** ^b^ ** + 2 days**	**PP 43** ^c^ ** + 28 days**	**PP 99** **-14 days, + 28 days**	**PP 120** ** ± 14 days**	**PP 127** ** ± 3 days**	**Dose 1 + 365 ± 28 days**	
**Visit type**	Screening	**Random-ization**	Safety	Safety	Safety	Safety and immuno	**DOSE 1 group B**	**DOSE 2 group B**	Safety and immuno	Safety	Safety and immuno	Early exit
Assessment of understanding	•											
Written informed consent (ICF) ^d^	•											
Inclusion/exclusion criteria	•	•										
Demographics ^**e**^	•											
Medical and obstetric history ^f^ and prestudy meds	•											
Physical examination ^f^	•						•	•				•
Obstetric exam ^f^	•	•				•						•^o^
Targeted physical examination ^f^		•	•	•	•				•	•	•	
Vital signs ^f^ incl. body temperature	•	•	•	•	•	•			•	•	•	•
Urine pregnancy test ^g^	•											
Hematology, chemistry ^g^	•											
Urine dipstick/urinalysis ^g^	•	•	•	•	•							
Syphilis, HIV, hepatitis B and C, malaria screening ^g^	•											
Obstetric ultrasound ^h^	•	•^h^			•							•^o^
Biometric scan		•	•	•	•	•	•	•	•	•	•	•
Contact information	•	•	•	•	•	•	•	•	•	•	•	•
Randomization		•										
Pre-vaccination symptoms^i^												
Vaccination							•	•				
30 min post-vaccination observation ^j^							•	•				
Adverse maternal, fetal outcomes		•	•	•	•	•	•					
SAE recording		•	•	•	•	•	•	•	•	•	•	•
Concomitant medications ^k^	•	•	•	•	•	•	•	•	•	•	•	•
Delivery history ^l^						•						
Post pregnancy physical examination ^l^						•						
Laboratory assessments ^m^						•						
Group B immunology subset supplemental procedures (*N* = 150)	**1**	**2**	**3**	**4**	**5**	**6**	**7**	**8**	**9**	**10**	**11**	EE
Humoral immunogenicity sample (serum)									•		•	
Breast milk sample (*N* = 10)												

pre-dose;  pre- and post-dose

^a^Visits 9 and 11 for group B immunology subset participants only

^b^PP1 refers to the day of pregnancy completion/termination. Visit 6 (PP1) should occur on the day of pregnancy completion/termination or within 2 days

^c^Visit 7 (PP 43) scheduled to occur at routine 6 week post-partum visit

^d^Must be signed before first study-related activity

^e^Maternal demographic information to be collected at screening includes: date of birth, race/ethnicity, education level, geographical location/residence, occupation, household size (number of persons living in the same home), and international travel history

^f^Information to be collected for medical and obstetric history, physical exams, and vital signs is specified in the protocol

^g^Urine dipstick for protein and glucose results will be performed at these visits. If results are 1 + or greater, then additional work-up for pre-eclampsia or gestational diabetes may be warranted

^h^Ultrasound not required at visit 2 if screening (visit 1) ultrasound completed within past 10 days and no other indication for ultrasound

^i^Investigator must check for acute illness or body temperature ≥ 38.0 °C at the time of vaccination. In such cases, the participant may be vaccinated at later time point

^j^Participants will be closely observed for a minimum of 30 min post-vaccination

^k^Includes any medical treatment/medications given to the mother during delivery (e.g., antibiotic prophylaxis)-specifying names of medications administered

^l^Information to be collected for delivery history and post-pregnancy examination is specified in the protocol

^m^Any routine laboratory assessment performed at time of delivery and other laboratory tests as medically indicated

^n^Visit 10: for women not part of the immunogenicity subset this visit will be the end of the trial

^o^Not required if participant is not pregnant

^p^If pregnancy completion/termination in a group B woman occurs prior to performing all in-pregnancy protocol visits, these missed visits will not be considered as protocol deviations

**Table 4:** Schedule of activities—women From Umurinzi

Visit #	1	2	3	4	5	EE
**Visit timing**		**Dose 2**	**Dose 2 + 28 days**	**Pregnancy completion/termination**	**PP 1 + 42 days**	**Early** **exit**
**Visit day and window**	** − 28 to 1**	**D57** ** − 14 days, + 28 days**	**D85 ± 7 days**	**PP 1** ^a^ ** + 2 days**	**PP 43** ^b^ ** ± 14 days**	
**Visit type**	Screening	**Dose 2**	Safety	Safety and immuno	Safety	Early exit
Assessment of understanding	•					
Written informed consent (ICF) ^c^	•					
Inclusion/exclusion criteria	•					
Demographics ^d^	•					
Medical and obstetric history ^e^; pre-pregnancy and pre-study meds	•					
Physical examination ^e^	•				•	•
Obstetric exam ^e^	•	•		•		•^m^
Targeted physical exam ^e^		•	•			
Vital signs ^e^ incl. body temperature	•		•	•	•	•
Urine pregnancy test ^f^	•					
Hematology, chemistry ^f^	•					
Urine dipstick/urinalysis ^f^	•	•	•			
Syphilis, HIV, hepatitis B and C, malaria screening ^f^	•					
Obstetric ultrasound ^g^	•		•			•^m^
Biometric scan	•	•	•	•	•	•
Contact information	•	•	•	•	•	•
Pre-vaccination symptoms ^h^						
Vaccination		•				
30 min post-vaccination observation^i^		•				
Adverse maternal, fetal outcomes		•	•	•	•	
SAE recording		•	•	•	•	•
Concomitant medications ^j^	•	•	•	•	•	•
Delivery history ^k^				•		
Post pregnancy physical examination ^k^				•		
Laboratory assessments ^l^				•		

pre-dose;  pre- and post-dose

^a^PP 1 refers to the day of pregnancy completion/termination. Visit 4 (PP 1) should occur on the day of pregnancy completion/termination or within 2 days

^b^Visit 5 (PP 43) scheduled to occur at routine 6 week post-partum visit

^c^Must be signed before first study-related activity

^d^Maternal demographic information to be collected at screening includes: date of birth, race/ethnicity, education level, geographical location/residence, occupation, household size (number of persons living in the same home), and international travel history

^e^Information to be collected for medical and obstetric history, physical exams, and vital signs is specified in the protocol

^f^Urine dipstick for protein and glucose will be performed at these visits. If results are 1 + or greater, additional work-up for pre-eclampsia and gestational diabetes may be warranted

^g^Ultrasound not required at visit 2 if screening (visit 1) ultrasound completed within past 10 days and no other indication for ultrasound

^h^Investigator must check for acute illness or body temperature ≥ 38.0 °C at the time of vaccination. In such cases, the participant may be vaccinated at later time point

^i^Participants will be closely observed for a minimum of 30 min post-vaccination

^j^Includes any medical treatment/medications given to the mother during delivery (e.g., antibiotic prophylaxis)-specifying names of medications administered

^k^Information to be collected for delivery history and post-pregnancy examination is specified in the protocol

^l^Any routine laboratory assessment performed at time of delivery and other laboratory tests as medically indicated

^m^Not required if participant is not pregnant

**Table 5:** Schedule of activities—neonates/infants from groups A, B, and Umurinzi

Visit #	1 ^a^	2 ^b^	3
**Visit timing**	**Pregnancy completion/termination**	**PP1 + 42 days**	**PP1 + 98 days**
**Visit day and window**	**PP 1** ** + 2 days**	**PP 43** ** ± 14 days**	**PP 99 ± 14 days**
**Visit type**	Safety	Safety	Safety and immuno
Fetal monitoring during labor^c^	•		
Birth outcome^d^	•		
Apgar score^e^	•		
Neonatal physical exam^f^	•		
Medication^g^	•	•	•
Nutrition^h^	•	•	•
Infant physical exam ^f^		•	•
Adverse neonatal/infant outcomes	•	•	•
SAE collection	•	•	•
Humoral immunogenicity sample (serum)^i^			•

^a^Visit 1 (PP 1) should occur on the day of pregnancy completion or within 2 days

^b^Informed consent for any study procedures to be performed for infants ≥ 28 days of age will be obtained from mother at enrolment

^c^Record key findings of fetal monitoring such as fetal position at delivery, gestational age, complications, fetal heart rate, and uterine contractions during labor

^d^Live birth, stillbirth, neonatal death, parity

^e^Measured at 1, 5, and 10 min

^f^Information to be collected for neonatal physical examination and infant physical examination is specified in the protocol

^g^Any medical or intervention given to the neonate (e.g., antibiotic treatment, exchange transfusion, intravenous fluids, steroids or other immunosuppressive therapies, herbal remedies, respiratory support)

^h^Type of feeds (e.g., breast milk (mother/donor), formula feeding, parenteral nutrition, mixed feeding) and their respective start and stop times should be recorded in months of age

^i^Blood sample (1 mL) of 75 infants born to women from the group A immunology subset

#### Sample size {14}

The primary outcomes of this unpowered study are adverse maternal, fetal, and neonatal/infant outcomes in vaccinated pregnant women and their neonates. The sample size was determined based on feasibility and estimated background incidence of the primary study outcomes. Background rates for adverse maternal and fetal outcomes in pregnancy are not comprehensively available for Rwanda for all events of interest. However, the Rwanda Demographic Health Survey (2014–2015) [16] provides rates of maternal death (210 per 100,000 live births) and neonatal death (20 per 1000 live births). With the proposed sample size of 1000, the probability of observing at least 1 AE occurring at a rate of 1/1000 is 63%. If no (S)AE (this includes adverse maternal/fetal outcomes) is observed in the vaccine group (*N* = 1000), this would provide 95% confidence that the true incidence is no more than 0.3%.

### Recruitment {15}

Pregnant women will be recruited from antenatal care (ANC) clinics. Recruitment will be conducted with residents of the catchment areas of the participating District Hospitals in Rwanda. Recruited women will be referred to the study sites located within the two District Hospitals for all study procedures.

## Assignment of interventions: allocation

### Sequence generation {16a}

Randomization will be used to minimize bias in the assignment of participants to group A or B, to increase the likelihood that known and unknown participant attributes are evenly balanced across both groups. An allocation template was created using Microsoft Excel. A total of 1051 sequentially numbered rows were created (1–1051), and for each row, a random number between 0 and 1 was generated. All rows with a random number less than or equal to 0.5 were allocated to group A and all rows with a random number greater than 0.5 were allocated to group B. Each site-specific set of 1051 sealed randomization envelopes will be securely stored at the appropriate site.

### Concealment mechanism {16b}

The allocation table is not visible to the trial staff conducting the randomization; therefore, the group assignment within each numbered envelope will be unknown to both participants and staff until each envelope is opened. Envelopes used for randomization are security-type (featuring a printed tint pattern inside the envelope to prevent contents from being read through a sealed envelope). The cards and envelopes will be prepared by the study team and inspected separately by 2 other members of the study team to ensure exact correspondence to the allocation table prior to sealing the envelopes and deploying them to the sites.

### Implementation {16c}

The allocation table was created by Dr. Susan Allen at Emory University. Trained study investigators or study staff will enroll participants. Participants will be invited to reach into the bin to select a single sealed envelope containing a randomization assignment at the time of enrollment. Study staff will supervise this process to ensure that only one envelope is selected and opened. Once the envelope has been opened and the randomization card revealed, the randomization assignment will be complete and irreversible. Randomization of subjects will be documented in the Redcap Cloud clinical database by study staff.

## Assignment of interventions: blinding

### Who will be blinded {17a}

There will be no blinding in this study. Since the study vaccines have been judged to be of potential benefit to non-pregnant adults in the community through the Umurinzi campaign, an open-label design was selected for this trial to minimize the delay to vaccination for the control group.

### Procedure for unblinding if needed {17b}

Not applicable as this is an open-label study.

## Data collection and management

### Plans for assessment and collection of outcomes {18a}

All study-related procedures among pregnant women and their newborns will be performed in District Hospitals including pre-enrolment screening and post-delivery evaluation of the mother and child. Study visits will be conducted at the same time as planned ANC visits when possible. Outcomes may be abstracted from medical records and women will be tracked over the study using a unique study ID combined with their District Hospital-assigned medical record ID and biometric data collection. All participating District Hospital units will have the appropriate training and diagnostic capability to capture trial endpoints. Data will be entered into RedCap Cloud collection forms.

### Plans to promote participant retention and complete follow-up {18b}

Study participants are pregnant women and they have regular clinic visits; therefore, there are no foreseen major issues with participant retention at this stage. The following study tools/materials/initiatives are available to further ensure participant retention:

Participants will be reimbursed for travel costs to and from the study site.

- Study visits and contacts will allow sites to develop and maintain a relationship with participants
- Participants are being mobilized from communities within the catchment area of the study site to make participant out-reach feasible
- Mobile messaging technology (MOTECH) will be utilized to stay in close contact with participants. This is automated phone messaging system that will use follow-up messages to participants and reminders of when to return to clinics for a visit
- Community engagement staff of study site will reach out proactively and directly to study participants for any missing visits

### Data management {19}

Steps to be taken to ensure the accuracy and reliability of data include the selection of qualified investigators and appropriate study sites, review of protocol procedures with the investigator and study-site personnel before the study, and periodic monitoring visits by the sponsor. Written instructions will be provided for collection, handling, storage, and shipment of samples. Guidelines for CRF completion will be provided and reviewed with study-site personnel before the start of the study.

The sponsor will review CRF for accuracy and completeness during on-site monitoring visits and after transmission to the sponsor; any discrepancies will be resolved with the investigator or designee, as appropriate. After the upload of the data into the study database, they will be verified for accuracy and consistency with the data sources. The Data Management Plan (DMP) details data entry and coding procedures, secure storage, data range checks for data values, manual data review procedures, source data verification, and system and user query generation and resolution guidelines.

### Confidentiality {27}

The collection and processing of personal data from participants enrolled in this study will be limited to those data that are necessary to fulfill the objectives of the study. These data will be collected and processed with adequate precautions to ensure confidentiality and compliance with applicable data privacy protection laws and regulations. Appropriate technical and organizational measures to protect the personal data against unauthorized disclosures or access, accidental or unlawful destruction, or accidental loss or alteration must be put in place. Sponsor personnel whose responsibilities require access to personal data agree to keep the identity of participants confidential.

The informed consent obtained from the participant includes explicit consent for the processing of personal data and for the investigator/institution to allow direct access to his or her original medical records (source data/documents) for study-related monitoring, audit, IEC/IRB review, and regulatory inspection. This consent also addresses the transfer of the data to other entities and to other countries.

### Plans for collection, laboratory evaluation, and storage of biological specimens for genetic or molecular analysis in this trial/future use {33}

Samples collected in this study may be stored for up to 15 years (or according to local regulations) for additional research. Samples will only be used to understand Ad26.ZEBOV and MVA-BN-Filo, to understand EVD, to understand differential intervention responders, and to develop tests/assays related to Ad26.ZEBOV and MVA-BN-Filo and EVD. The research may begin at any time during the study or the post-study storage period. Stored samples will be coded throughout the sample storage and analysis process and will not be labeled with personal identifiers. The informed consent obtained from the participant includes information on the time period that samples will be stored. Participants may withdraw their consent for their samples to be stored for research.

## Statistical methods

### Statistical methods for primary and secondary outcomes {20a}

For purposes of analysis, the following populations are defined:

- Full analysis set (FAS): The FAS will include all randomized participants. All safety analyses will be made on the FAS
- Per-protocol immunogenicity (PPI): The PPI population will include all randomized and vaccinated participants for whom immunogenicity data are available excluding participants with major protocol deviations expecting to impact the immunogenicity outcomes

Of note, women enrolled from Umurinzi will be analyzed separately. No formal statistical testing is planned. The number and percentage of participants screened, enrolled, randomized, receiving two vaccine doses, and completing follow-up will be summarized by study arm.

Baseline demographic characteristics (including gestational age and obstetric risk assessment) will be tabulated overall and by study arm.

The frequency and percentage of women for whom at least one of the indicated adverse outcomes was reported as a primary endpoint (see above) will be summarized by study group. For each separate adverse maternal/fetal and adverse neonatal/infant outcome, the number and percentage of participants who experience at least 1 occurrence of the given event will also be tabulated by study group (groups A and B, and their infants). The verbatim terms used in the CRF by investigators to identify AEs will be coded using the Medical Dictionary for Regulatory Activities (MedDRA). The reporting of the categorical safety data will include the incidence, severity, relatedness, and type of AEs. Continuous safety parameters will be analyzed descriptively (showing the mean, standard deviation, median, and quartiles [Q1 and Q3]).

For participants from group A, all reported AEs, events-related diary information (solicited local at injection site and systemic, and unsolicited) with onset within 4 weeks post dose 1 or dose 2 vaccination (i.e., treatment-emergent AEs) will be included in the analysis. For each AE, the number and percentage of participants who experience at least 1 occurrence of the given event will be summarized. Of note, solicited and unsolicited AEs will not be collected for group B participants.

Summaries, listings, datasets, or participant narratives may be provided, as appropriate, for those participants who die, who discontinue intervention due to an AE, or who experience a severe or a serious AE.

For participants from group A, solicited local (at injection site) and systemic AEs will be summarized descriptively. The overall frequencies per as well as frequencies according to severity and duration will be calculated for solicited AEs. In addition, the number and percentages of participants with at least one solicited local (at injection site) or systemic AE will be presented. Frequencies of unsolicited AEs, separately for all and vaccination-related only, will be presented by System Organ Class and preferred term, while those of solicited AEs will be presented only by preferred term.

Laboratory data will be listed by type of laboratory test. Reference ranges and markedly abnormal results will be used in the listing of laboratory data. Laboratory analyses will not be conducted routinely after screening.

Vital signs including temperature, pulse/heart rate, and blood pressure (systolic and diastolic) will be summarized over time, using descriptive statistics and/or graphically. The percentage of participants with values beyond clinically important limits will be summarized.

Physical examination findings will be listed.

The immunogenicity analyses will be performed on the PPI set. Descriptive statistics of the anti-EBOV GP binding antibody (including responder rate, geometric means with their corresponding 95% CIs, median, interquartile range, minimum, maximum, as applicable) will be calculated for continuous immunologic parameters at each time point. Graphical representations of immunologic parameters will be prepared, as applicable. Frequency tabulations will be calculated for discrete (qualitative) immunologic parameters at each time point.

Detailed statistical methodology for all analyses are described in the statistical analysis plan (SAP).

### Interim analyses {21b}

There are no planned interim analyses. If required, an interim analysis may be performed during the study for the purpose of informing future vaccine-related decisions in a timely manner. Study pausing rules, including who reviews reasons for study pause and makes determinations regarding the decision to terminate the trial, are described below.

### Methods for additional analyses (e.g., subgroup analyses) {20b}

No additional analyses are planned.

### Methods in analysis to handle protocol non-adherence and any statistical methods to handle missing data {20c}

The safety analysis will be done on the full analysis dataset. No data will be imputed.

### Plans to give access to the full protocol, participant level-data and statistical code {31c}

The protocol itself is shared with this manuscript. Janssen has an agreement with the Yale Open Data Access (YODA) Project to serve as the independent review panel for evaluation of requests for CSRs and participant level data from investigators and physicians for scientific research that will advance medical knowledge and public health. Data will be made available following publication and approval by YODA of any formal requests with a defined analysis plan. For more information on this process or to make a request, please visit The Yoda Project site at http://yoda.yale.edu. The data sharing policy of Janssen Pharmaceutical Companies of Johnson & Johnson is available at https://www.janssen.com/clinical-trials/transparency.

## Oversight and monitoring

### Composition of the coordinating center and trial steering committee {5d}

The Center for Family Health Research (CFHR), led by the principal investigator, Dr. Etienne Karita, in Kigali, Rwanda, is coordinating the implementation of this trial in the participating country of Rwanda. The trial steering committee comprises representatives from CFHR, Emory University, Janssen Vaccines and Prevention (regulatory sponsor), and the Coalition for Epidemic Preparedness and Innovation (CEPI)-the trial funder.

### Composition of the data monitoring committee, its role and reporting structure {21a}

An independent data monitoring committee (IDMC) will be established to review aggregated safety data and to ensure the continuing safety of the participants enrolled in this study. This committee will consist of at least one medical expert in each of the relevant therapeutic areas of obstetrics and pediatrics and at least one statistician; committee membership responsibilities, authorities, and procedures are documented in its Charter. All members are independent from the sponsor and have no competing interests. The committee will meet periodically to review all available aggregated safety data at that point in time. After the review, the IDMC will make recommendations regarding the continuation of the study.

The IDMC will review safety data related to a study pause and provide a written recommendation to the sponsor as to whether study activities, including vaccination, should be resumed.

Vaccinations for an individual participant may be suspended for safety concerns other than those described in the pausing criteria, at the discretion of the investigator if he/she feels the participant’s safety may be threatened. The sponsor’s medical monitor or designee or the investigator(s) (upon consultation with the sponsor’s medical monitor or designee) may initiate IDMC review for any single event or combination of multiple events which, in their professional opinion, could jeopardize the safety of the participants or the reliability of the data. Vaccinations for the study may be suspended for safety concerns other than those described above, or before pausing rules are met, if, in the judgment of the IDMC, participant safety may be threatened.

#### Adverse event reporting and harms {22}

If a serious and unexpected AE occurs for which there is evidence suggesting a causal relationship between the study vaccine and the event (e.g., death from anaphylaxis), the event must be reported as a serious and unexpected suspected adverse reaction even if it is a component of the study endpoint (e.g., all-cause mortality). All AEs, regardless of seriousness, severity, or presumed relationship to study vaccine, must be recorded using medical terminology in the source document and the CRF. Whenever possible, diagnoses should be given when signs and symptoms are due to a common etiology (e.g., cough, runny nose, sneezing, sore throat, and head congestion should be reported as "upper respiratory infection"). Investigators must record in the CRF their opinion concerning the relationship of the AE to study therapy. All measures required for AE management must be recorded in the source document and reported according to sponsor instructions. For all studies with an outpatient phase, including open-label studies, the participant may be provided with a "wallet (study) card" and instructed to carry this card with them for the duration of the study indicating the following:

- Study number
- Statement, in the local language(s), that the participant is participating in a clinical study
- Investigator’s name and 24-h contact telephone number
- Local sponsor’s name and 24-h contact telephone number (for medical personnel only)
- Site number
- Participant number
- Any other information that is required to do an emergency breaking of the blind

All SAEs that have not resolved by the end of the study, or that have not resolved upon discontinuation of the participant's participation in the study, must be followed until any of the following occurs:

- The event resolves
- The event stabilizes
- The event returns to baseline, if a baseline value/status is available
- The event can be attributed to agents other than the study vaccine or to factors unrelated to study conduct
- It becomes unlikely that any additional information can be obtained (participant or health care practitioner refusal to provide additional information, lost to follow-up after demonstration of due diligence with follow-up efforts)

Suspected transmission of an infectious agent by a medicinal product will be reported as a SAE. Any event requiring hospitalization (or prolongation of hospitalization) that occurs during the course of a participant's participation in a study must be reported as a SAE, except hospitalizations for the following:

- Hospitalization for normal pregnancy completion (i.e., delivery)
- Hospitalizations not intended to treat an acute illness or AE (e.g., social reasons such as pending placement in long-term care facility)
- Surgery or procedure planned before entry into the study (must be documented in the CRF). Note: Hospitalizations that were planned before the signing of the ICF, and where the underlying condition for which the hospitalization was planned has not worsened, will not be considered SAEs. Any AE that results in a prolongation of the originally planned hospitalization is to be reported as a new SAE.

The cause of death of a participant in a study, whether or not the event is expected or associated with the study vaccine, is considered a SAE.

### Frequency and plans for auditing trial conduct {23}

Representatives of the sponsor’s clinical quality assurance department may visit the study site at any time during or after completion of the study to conduct an audit of the study in compliance with regulatory guidelines and company policy. These audits will require access to all study records, including source documents, for inspection. Participant privacy must, however, be respected. The investigator and study-site personnel are responsible for being present and available for consultation during routinely scheduled study-site audit visits conducted by the sponsor or its designees. Similar auditing procedures may also be conducted by agents of any regulatory body, either as part of a national GCP compliance program or to review the results of this study in support of a regulatory submission. The investigator should immediately notify the sponsor if he or she has been contacted by a regulatory agency concerning an upcoming inspection.

### Plans for communicating important protocol amendments to relevant parties (e.g., trial participants, ethical committees) {25}

Neither the investigator nor the sponsor will modify this protocol without a formal amendment by the sponsor. All protocol amendments must be issued by the sponsor and signed and dated by the investigator. Protocol amendments must not be implemented without prior IEC/IRB approval, or when the relevant competent authority has raised any grounds for non-acceptance, except when necessary to eliminate immediate hazards to the participants, in which case the amendment must be promptly submitted to the IEC/IRB and relevant competent authority. Documentation of amendment approval by the investigator and IEC/IRB must be provided to the sponsor. When the change(s) involve only logistic or administrative aspects of the study, the IEC/IRB (where required) only needs to be notified.

During the course of the study, in situations where a departure from the protocol is unavoidable, the investigator or other physician in attendance will contact the appropriate sponsor representative listed in the Contact Information page(s), which will be provided as a separate document. Except in emergency situations, this contact should be made before implementing any departure from the protocol. In all cases, contact with the sponsor must be made as soon as possible to discuss the situation and agree on an appropriate course of action. The data recorded in the CRF and source documents will reflect any departure from the protocol, and the source documents will describe this departure and the circumstances requiring it.

### Dissemination plans {31a}

Consistent with Good Publication Practices and International Committee of Medical Journal Editors (ICMJE) guidelines, the sponsor shall have the right to publish such primary (multicenter) data and information without approval from the investigator. The investigator has the right to publish study site-specific data after the primary data are published. If an investigator wishes to publish information from the study, a copy of the manuscript must be provided to the sponsor for review at least 60 days before submission for publication or presentation. Expedited reviews will be arranged for abstracts, poster presentations, or other materials. If requested by the sponsor in writing, the investigator will withhold such publication for up to an additional 60 days to allow for filing of a patent application. In the event that issues arise regarding scientific integrity or regulatory compliance, the sponsor will review these issues with the investigator. The sponsor will not mandate modifications to scientific content and does not have the right to suppress information. Authorship of publications resulting from this study will be based on the guidelines on authorship, such as those described in the ICMJE Recommendations for the Conduct, Reporting, Editing and Publication of Scholarly Work in Medical Journals, which state that the named authors must have made a significant contribution to the conception or design of the work; or the acquisition, analysis, or interpretation of the data for the work; and drafted the work or revised it critically for important intellectual content; and given final approval of the version to be published; and agreed to be accountable for all aspects of the work in ensuring that questions related to the accuracy or integrity of any part of the work are appropriately investigated and resolved.

#### Public and patient involvement in the protocol

No members of the public or patient populations were involved in the design of this protocol.

## Discussion

Some practical and operational issues warrant discussion. First, efforts will be made to ensure that the reference ranges for the various laboratory measures are relevant in the Rwandan context. The reference ranges in this protocol are broad as many reference ranges are unknown in the Rwandan context. These can be modified based on the reference ranges used locally at the study site laboratories. Another practical issue is determining gestational age and estimated date of delivery. We will develop procedures for determining a “consensus” estimated date of delivery based on last menstrual period and ultrasound. Though this is not a blinded trial, it is notable that labor and delivery staff are not associated with the trial and do not know which arm the women are in. This may prevent some bias when assessing maternal and newborn outcomes. The decision to potentially allow enrollment of women during their first trimester of pregnancy after rigorous safety data review by medical officers and the IDMC may provide additional, novel data that supports vaccinating and protecting women and their fetuses as early in pregnancy as possible. We expect the proportion of women recruited into the study during their first trimester to be between 1.5% (assuming the IDMC does not open recruitment to this population) and 4.25% (assuming the IDMC does open recruitment to this population and based on an expected rate of enrollment of this group beginning half-way through the study). Finally, this trial is being conducted during the COVID-19 pandemic. Some COVID-19 symptoms may be similar to vaccine reactogenicity. Additionally, nationwide restrictions due to COVID-19 may necessitate some remote data collection and could impact site monitor visitation schedules.

## Trial status

Protocol version number and date: July 8, 2020 v2.0

Date recruitment began: 6 October 2020.

Approximate date when recruitment will be completed: January 2022. 

## Data Availability

When preparing for the manuscript describing the results of the study, there will measures in place to allow all authors to access the study database, should they wish to do so. The manuscript authors who accessed and verified the data have been indicated.

## Abbreviations

AE: Adverse event
ANC: Antenatal clinic
AOU: Assessment of understanding
ART: Antiretroviral therapy
CRF: Case report form(s) (paper or electronic as appropriate for this study)
DMP: Data Management Plan
DRC: Democratic Republic of Congo
EMA: European Medicines Agency
EVD: Ebola virus disease
FDA: Food and Drug Administration
IB: Investigator’s Brochure
IDMC: Independent Data Monitoring Committee
IEC: Independent Ethics Committee
eDC: Electronic data capture
GCP: Good Clinical Practice
GMC: Geometric Mean Concentration
HBsAg: Hepatitis B surface antigen
HCV: Hepatitis C virus
HITT: Heparin-induced thrombocytopenia and thrombosis
HIV: Human immunodeficiency virus
IAC: Interim Analysis Committee
ICF: Informed consent form
ICH: International Conference on Harmonisation
IEC: Independent Ethics Committee
IM: Intramuscular
ICMJE: International Committee of Medical Journal Editors
IMP: Investigational Medicinal Product
IPPI: Investigational Product Preparation and Administration Instructions
IRB: Institutional Review Board
MedDRA: Medical Dictionary for Regulatory Activities
MRU: Medical resource utilization
NIMP: Non-Investigational Medicinal Product
PMTCT: Prevention of mother-to-child transmission
SAE: Serious adverse event
SAP: Statistical analysis plan
SIPPM: Site Investigational Product Procedure Manual
SoA: Schedule of activities
SUSAR: Suspected unexpected serious adverse reaction
TTS: Thrombotic thrombocytopenia syndrome
USP: United States Pharmacopeia
VISP: Vaccine-induced seropositivity
WHO: World Health Organization
